# Supplementary material for: Risk factors of hepatocellular carcinoma in type 2 diabetes patients: A two-centre study in a developing country
Source: PLoS One. 2021 Dec 9;16(12):e0260675. doi: 10.1371/journal.pone.0260675 (PMC8659343; doi:10.1371/journal.pone.0260675)
Supplement: S1 Appendix — (DOCX) [file pone.0260675.s001.docx]

Missing data analysis.

The Little’s MCAR test was performed with p-value= 0.267, which indicates that the missing data is at random [1]. Thus, multiple imputation method was applied to these variables prior to the inferential analysis.


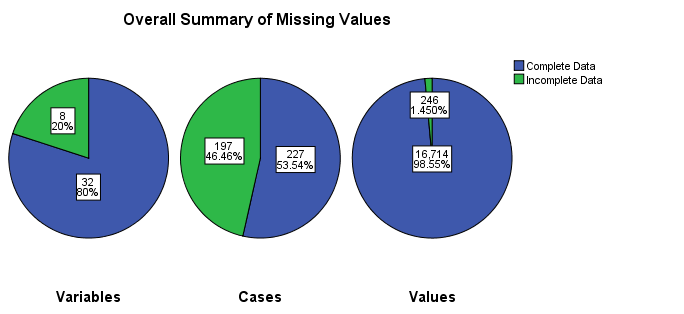


Figure 1 Missing data, based on the variables, cases and values

Table 1 Percentage of missing values according to variables

| Variables | Missing | | Valid |
| --- | --- | --- | --- |
|  | n | % | N |
| International normalized ratio, INR | 78 | 18.4 | 346 |
| Mean platelet volume, MPV | 68 | 16.0 | 356 |
| Duration of DM | 46 | 10.1 | 381 |
| Albumin globulin ratio, AGR | 26 | 6.1 | 398 |
| Total bilirubin | 18 | 4.2 | 406 |
| Alanine transaminase (ALT) | 6 | 1.4 | 418 |
| Hemoglobin-A1c, HbA1c | 4 | 0.9 | 420 |
| Alkaline phosphatase (ALP), | 3 | 0.7 | 421 |

Table 2 : The comparison of incomplete original data and imputed dataset for the affected variables

| **Risk factors** | | **Original data** | | | | **Imputed data (N=424)** | | |
| --- | --- | --- | --- | --- | --- | --- | --- | --- |
|  |  | **Valid N** | **Cases, n (%)** | **Controls, n (%)** | **p-value^a^** | **Cases, n (%)** | **Controls, n (%)** | **p-value^a^** |
| ***DM-related characteristic*** | |  |  |  |  |  |  |  |
| Duration of T2DM, years | | 378 |  |  | 0.023 |  |  | 0.051 |
|  | ≥10 |  | 81 (48.5) | 127(60.2) |  | 104 (49.1) | 128 (60.4) |  |
|  | 0–9 |  | 86 (51.5) | 84(39.8) |  | 108 (50.9) | 84 (39.6) |  |
| **Biochemical profile** | |  |  |  |  |  |  |  |
| ***DM monitoring*** | |  |  |  |  |  |  |  |
| HbA1c, % | | 420 |  |  | 0.650 |  |  | 0.689 |
|  | ≥8.5 |  | 81 (38.9) | 78 (36.8) |  | 82 (38.7) | 78 (36.7) |  |
|  | <8.5 |  | 127 (61.1) | 134 (63.2) |  | 130 (61.3) | 134 (63.3) |  |
| ***Full blood count*** | |  |  |  |  |  |  |  |
| MPV, fL | | 356 |  |  | 0.062 |  |  | 0.183 |
|  | >11 |  | 67 (35.8) | 45 (26.6) |  | 79 (37.3) | 66 (31.1) |  |
|  | ≤11 |  | 120 (64.2) | 124 (73.4) |  | 133 (62.7) | 146 (68.9) |  |
| ***Liver function test*** | |  |  |  |  |  |  |  |
| AGR | | 398 |  |  | 0.022 |  |  | 0.048 |
|  | <1.1 |  | 140 (74.5) | 134 (63.8) |  | 154 (72.6) | 135 (63.7) |  |
|  | ≥1.1 |  | 48 (25.5) | 76 (36.2) |  | 58 (27.4) | 77 (36.3) |  |
| TBil, µmol/L | | 406 |  |  | <0.001 |  |  | <0.001 |
|  | >21 |  | 72(35.8) | 25 (12.2) |  | 79 (37.3) | 30 (14.2) |  |
|  | ≤21 |  | 129 (64.2) | 180 (87.8) |  | 133 (62.7) | 182 (85.8) |  |
| ALP, IU/L | | 421 |  |  | <0.001 |  |  | <0.001 |
|  | >129 |  | 96 (45.7) | 48 (22.7) |  | 98 (46.2) | 48 (22.6) |  |
|  | ≤129 |  | 114 (54.3) | 163 (77.3) |  | 114 (53.8) | 164 (77.4) |  |
| ALT, IU/L | | 418 |  |  | <0.001 |  |  | <0.001 |
|  | ≥25 |  | 158(76.3) | 86(40.8) |  | 162 (76.4) | 87 (41.0) |  |
|  | <25 |  | 49(23.7) | 125 (59.2) |  | 50 (23.6) | 125 (59.0) |  |
| ***Coagulation profile*** | | |  |  |  |  |  |  |
| INR | | 346 |  |  | 0.104 |  |  | 0.506 |
|  | >1.2 |  | 40 (23.1) | 28 (16.2) |  | 58 (27.4) | 52 (24.5) |  |
|  | ≤1.2 |  | 133 (76.9) | 145(83.8) |  | 154 (72.6) | 160 (75.5) |  |

^a^ Chi-square tests

1. Tabachnick BG, Fidell LS. Using Multivariate Analysis. New York: Pearson publishers; 2013.
